# Associations Between Chronic Irritability and Sensory Processing Difficulties in Children and Adolescents

**DOI:** 10.3389/fpsyt.2022.860278

**Published:** 2022-04-28

**Authors:** Yuji Harima, Dai Miyawaki, Ayako Goto, Kaoru Hirai, Shoko Sakamoto, Hiroki Hama, Shin Kadono, Sayaka Nishiura, Koki Inoue

**Affiliations:** ^1^Department of Neuropsychiatry, Osaka Metropolitan University Graduate School of Medicine, Osaka, Japan; ^2^Department of Pediatrics, Osaka Metropolitan University Graduate School of Medicine, Osaka, Japan

**Keywords:** chronic irritability, mood dysregulation, sensory processing difficulties, sensory over-responsivity, short sensory profile, children behavior checklist (CBCL)

## Abstract

Irritability is one of the most common reasons for which children and adolescents are referred for psychiatric evaluation and care. However, clinical irritability is difficult to define; thus, its prevalence varies widely. Chronic irritability may be associated with sensory processing difficulties (SPD), but little is known about the relationship between these two factors in clinical populations. In this study, we examined the prevalence of chronic irritability and its association with SPD in 166 children aged 5–16 years who were referred to the psychiatric outpatient clinic of the Osaka City University Hospital. Chronic irritability and parent-reported scores for the Short Sensory Profile, Infant Behavior Checklist-Revised, Child Behavior Checklist, and Kiddie Schedule for Affective Disorders and Schizophrenia for School-Age Children (Present and Lifetime version) questionnaires were used for assessment. A total of 22 children (13.2%) presented with chronic irritability (i.e., the irritability group) and were more likely to have oppositional defiant disorder, externalizing problems, and attention issues than those without chronic irritability (i.e., the control group). SPD were reported in eight (36%) patients in the irritability group and in 21 (15%) in the control group (*p* = 0.029). Moreover, compared to the control group, the irritability group showed a significant difference in almost all items of the Short Sensory Profile. Chronic irritability was associated with more severe overall SPD, even after adjusting for possible confounding factors (internalizing and externalizing problems, age, sex, and low income). We provide evidence to support our hypothesis that chronic irritability is associated with SPD in children and adolescents. Therefore, SPD should be assessed to provide appropriate interventions in children and adolescents with chronic irritability.

## Introduction

Irritability is one of the most common reasons for which children and adolescents are referred for psychiatric evaluation and care (1). Irritability is present in numerous disorders, including autism spectrum disorder (ASD), oppositional defiant disorder (ODD), attention-deficit hyperactivity disorder (ADHD), anxiety disorders, major depressive disorder, and bipolar disorder (2–8). Although several questionnaires exist to assess irritability (9), its definition is difficult, and thus its prevalence varies widely. A recent community-based evaluation showed that 99.3% of individuals experienced at least one incident of phasic or chronic irritability through their lifetime, indicating that irritability is a common psychiatric symptom (10). Contrastingly, Althoff et al. found that only 0.1% of adolescents had severe irritability (though this finding was based on the strictest frequency criteria) (11). In the International Classification of Diseases 11th Revision (ICD-11), chronic irritability is listed as a symptom of disruptive behavior or dissocial disorders, while in the Diagnostic and Statistical Manual of Mental Disorders, Fifth Edition (DSM-5), it is included in both the disruptive, impulse control, and conduct disorder category as well as the depressive disorders section (12). This discrepancy between the ICD-11 and the DSM-5 may reflect the difficulty in classifying chronic irritability (12). On the other hand, the validity of distinguishing between phasic and chronic irritability has been empirically supported (13–15). Children and adolescents with chronic irritability not only have externalizing problems (13, 14), but are also at increased risk for academic struggles, poverty, suicidality (16), unipolar depression, and anxiety disorders later in life (13, 14, 16–21).

Chronic irritability has recently been suggested to be associated with sensory processing difficulties (SPD) (22). Namely, Benarous et al. reported that children with chronic irritability and temper outbursts exhibited more severe SPD than did typically developing children (22). Meanwhile, SPD is included in the diagnostic criteria for ASD and is prevalent in neurodevelopmental disorders, including ASD and ADHD (23–26). Previous studies have also shown that SPD is associated with internalizing (27) and externalizing problems (28, 29); co-occurring disorders such as schizophrenia (30, 31), anxiety disorders, stress-related disorders (32, 33), sleep disturbances (34), and anorexia nervosa (35); and lower age, lower baseline socioeconomic status, and male sex (36, 37). A study in a recent epidemiological survey found that an estimated 8% of school-aged children have experienced SPD (38). These studies suggest that SPD is associated with a variety of mental disorders and not just with ASD. However, little is known about the relationship between chronic irritability and SPD in clinical populations. Moreover, no studies have confirmed this relationship after adjusting for comorbidities and socioeconomic variables, which may act as confounders.

The aims of our study were: (a) to examine the prevalence of chronic irritability as defined by the degree, duration, and frequency of symptoms, and its correlation with comorbidities and psychopathologies in a clinical psychiatric population and (b) to test our hypothesis that chronic irritability is associated with SPD.

## Materials and Methods

### Participants

**FIGURE 1:**
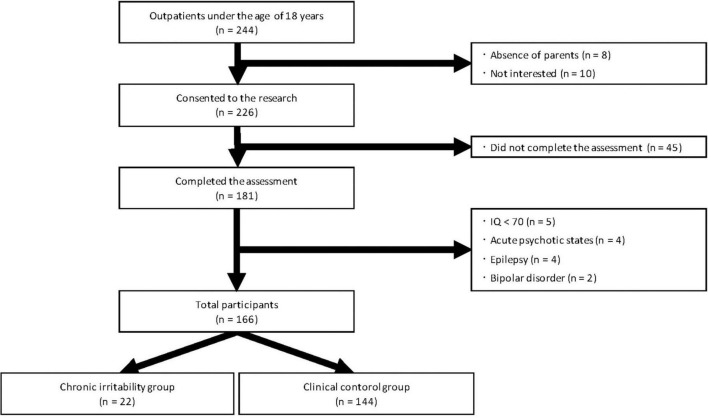
Flow chart of the patient selection process.

A summary of the study enrollment process is presented in Figure 1. The eligible study subjects were 244 children aged 5–17 years who were consecutively referred to the psychiatric outpatient clinic of the Osaka City University Hospital (Osaka, Japan) between April 2018 and April 2021. The participants visited the clinic for at least 3 months and were assessed by a multidisciplinary team that included expert child psychiatrists, psychologists, and psychiatric social workers. We excluded children whose parents were chronically absent or unavailable (*n* = 8) and those whose parents did not consent to their children participating in our study (*n* = 10). We also excluded children who refused to participate in semi-structured interviews or who did complete all assessments (*n* = 45). Children with intellectual disabilities (*n* = 5) (IQ < 70 based on the Wechsler Intelligence Scale for Children-Third or Fourth Edition; WISC-III or -IV) in whom symptom evaluation was difficult, those experiencing acute psychotic states (*n* = 4), those with severe neurological impairments or refractory epilepsy (*n* = 4), and those with bipolar disorder (*n* = 2) were also excluded. The remaining 166 children (aged 5–16 years) were divided into two groups: 22 with and 144 without chronic irritability.

Socioeconomic status has been shown to affect a variety of brain regions, including those associated with language, executive function, and attention, and is also strongly associated with health status (39, 40). Therefore, we conducted interviews to collect information on (1) parental absence (i.e., information on the absence of either the father or the mother), (2) family income, and (3) years of parental education. With regard to family income, we categorized households receiving public assistance or with an annual income of <3 million yen as low income.

We obtained written informed consent from all children and their parents before participation. The research protocol was reviewed and accepted by the Ethical Committee of Osaka City University Graduate School of Medicine. The research was performed according to the principles of the Declaration of Helsinki and its later amendments.

### Measurement

A multidisciplinary team converged on a diagnosis by integrating the information from the following diagnostic interviews and questionnaires.

#### Definition and Assessment of Chronic Irritability

For this study, we developed a new semi-structured interview that was administered to the children and their parents separately to assess present chronic irritability. The interview includes the following questions: (1) “Do you feel irritable?” (2) “Have you felt this way almost every day for the past year?” (3) “Is there a period of time, as long as a couple of months, when you didn’t feel this way?” (4) “Do you feel irritable throughout most of the day?” (5) “In how many situations do you feel irritable, at home, at school, with other children?” (6) “Do your parents, teachers, or other children notice your feelings?” We defined chronic irritability as a chronic, persistently irritable mood lasting for at least 1 year in at least two settings. Moreover, this irritability must be present for most of the day nearly every day, and must be noticeable by others in the child’s environment. Our definition of chronic irritability is consistent with the definition of irritable mood within the diagnostic criteria for disruptive mood dysregulation disorder (DMDD) in the DSM-5 (41). When there were discrepancies between a child’s and parent’s report, we queried the child and parent about the incongruent information. When the disagreement was not resolved, we obtained additional information from the teachers. We examined the test–retest and inter-rater reliability of the first battery of interviews by re-obtaining participant answers two months later.

#### Child Behavior Checklist

In this study, the standard Japanese version of the Child Behavior Checklist (CBCL) was administered to the parents of the affected children as a broad psychopathological assessment. The CBCL is a parent-reported rating scale developed by Achenbach and Dumenci consisting of 113 questions that assess childhood psychopathology (42). The response scale for each question is as follows: 0, not true (as far as the proband knows); 1, somewhat or sometimes true; and 2, very often or often true. The scale is composed of three domains (total, internalizing, and externalizing scores) and eight subscales (withdrawal, somatic complaints, anxiety/depression, social problems, thought problems, attention problems, delinquent behavior, and aggressive behavior).

#### Short Sensory Profile and Sensory Processing Difficulties

To evaluate SPD, we used the standardized Japanese version of the Short Sensory Profile (SSP), which was administered by the children’s parents. The SSP is a 38-item parent-completed questionnaire created to determine the functional behaviors associated with SPD in children aged 3–10 years within seven sections: tactile sensitivity, taste/smell sensitivity, movement sensitivity, underresponsive/seeks sensation, auditory filtering, low energy/weak, and visual/auditory sensitivity. The SSP asks parents to respond to behavioral descriptions of various sensory-laden events using a 5-point Likert scale (43). The Japanese version of the SSP is an almost faithful translation of the original, although some changes have been made to account for cultural differences. In the Japanese version, the target age range was expanded to over 3 years, with higher scores indicating lower adaptive functioning and a higher frequency of responses.

When a child presents with an SSP total score in the “definite difference” range (scores > 2 standard deviations above the mean), it indicates that the child’s performance is among the lowest 2% of the research sample when compared to the sample of children without disabilities. Based on a previous study (44), we defined a child as having SPD if their SSP total score was within the “definite difference” range.

#### Kiddie Schedule for Affective Disorder and Schizophrenia

The Kiddie Schedule for Affective Disorder and Schizophrenia (Present and Lifetime version; K-SADS-PL), a semi-structured diagnostic interview, was administered to all parents by a single study investigator. The K-SADS-PL psychometric properties have been estimated as excellent in prior work, with high inter-rater reliability as indicated by a κ of 0.93 and high test–retest reliability as indicated by intraclass correlation coefficients ranging from 0.74–0.90 (45). Each item is determined as present, absent, or unknown. The diagnostic algorithm was implemented following international guidelines. This diagnostic interview has consistently demonstrated good inter-rater reliability and high concurrent validity across studies (46).

#### Infant Behavior Checklist-Revised

The Infant Behavior Checklist-Revised (IBC-R) is a 24-item parent-completed questionnaire used to assess traits of autism in infancy, and it is often used for children and adolescents older than two years of age (47, 48). All 24 items are evaluated on a two-point scale and higher scores indicate more problematic behavior. The IBC-R was developed by Kanai et al. (49). They administered the scale to children referred to a psychiatry outpatient clinic (autism sample, *N* = 68; non-autism sample, *N* = 63; males, *N* = 97; females, *N* = 34; mean age, 4.1 years; standard deviation, 2.2; age range, 0.8–13.7 years). They found that a cutoff score of 7 or more had a 16.9% false negative rate and 15% false positive rate. This analysis suggests that the optimal cutoff score is 7. A total score of 7 or more is indicative of clinically significant autism traits. Psychometric analysis indicated adequate content validity and concurrent validity. Internal consistency was moderate (49).

### Statistics

Comparisons were conducted between participants who did or did not present with chronic irritability (*n* = 22 vs. *n* = 144). Descriptive statistics (means, standard deviations, medians, ranges, and proportions) were calculated for demographic and clinical variables. Student’s *t*-tests or Mann–Whitney U tests were used to compare continuous variables as appropriate (i.e., depending on whether the variables followed a normal distribution). Pearson’s χ^2^ tests and Fisher’s exact tests (implemented when expected values were <5) were used to compare categorical variables. To examine the correlation between chronic irritability and SPD, a forced entry multiple regression analysis was performed with total SSP scores as the dependent variables. The presence of chronic irritability was used as the primary independent variable, and we adjusted for covariates (low income, sex, age at consultation, IBC-R score, CBCL internalizing, and externalizing scores) that have been reported to be associated with SPD and were considered potential confounders herein. Categorical variables (including chronic irritability, low income, and sex) were converted to dummy variables by creating vectors of ones and zeros (one if the characteristic existed or to indicate male sex, and zero otherwise). All data were analyzed with the Statistical Package for Social Sciences (SPSS) statistical software (version 26.0.0; SPSS Japan, Inc., Tokyo, Japan). A two-sided *p*-value of <0.05 was considered to be statistically significant for all tests.

## Results

Concerning the semi-structured interview that we created to assess chronic irritability, the test–retest reliability was high, with a kappa coefficient of 0.818 (*n* = 66) within an average 57-day interval and an inter-rater reliability of 0.806 (*n* = 13) within an average 63-day interval. A total of 22 children (13.3%) displayed chronic irritability. Table 1 presents the comparison of the sociodemographic characteristics, clinical features, and comorbidities of the two groups. No significant difference was found between the groups in terms of gender, years of parental education, parental absence, and low income. There were no significant differences in the IBC-R scores or percentage of children above the cutoff threshold between the two groups. The rates of disruptive behavior disorder (DBD) and ODD were significantly higher in the chronic irritability group than in the clinical control group (45.5 vs. 22.2%, *p* = 0.020; 45.5 vs. 20.1%, *p* = 0.009).

**TABLE 1:** Participant sociodemographic and clinical characteristics.

	Total		Chronic irritability group		Clinical control group		χ^2^/U	*p*
	(*n* = 166)		(*n* = 22)		(*n* = 144)			
**Sociodemographic and social characteristics**								
Gender, male, n (%)	95	(57.2)	12	(54.5)	83	(57.6)	0.075	0.785^a^
Age (year), mean (SD)	11.8		11.7	(2.7)	11.8	(2.6)	1587.5	0.987^b^
Years of parental education (year), mean (SD)	14.1		14.3	(1.5)	14.1	(1.7)	1229.5	0.664^b^
Parental absence, *n* (%)	33	(19.9)	6	(27.3)	27	(18.8)		0.390^c^
Low income, *n* (%)	48	(22.9)	6	(27.3)	32	(22.2)	0.276	0.599^b^
**Infant Behavior Checklist-Revised**								
IBC-R score, mean	2.4		4.1	(6.0)	2.1	(3.5)	1770.5	0.384^b^
IBC-R, number over the cutoff point, *n* (%)	25	(15.1)	5	(22.7)	20	(13.9)		0.334^c^
**K-SADS-PL diagnoses**								
Mood disorders, *n* (%)	53	(31.9)	6	(27.3)	47	(32.6)	0.253	0.615^a^
Major depressive disorder, *n* (%)	18	(10.8)	2	(9.1)	16	(11.1)		1.000^c^
Dysthymia, *n* (%)	4	(2.4)	0	(0)	4	(2.8)		1.000^c^
Adjustment disorder, *n* (%)	31	(18.7)	4	(18.2)	27	(18.8)		1.000^c^
Anxiety disorders, *n* (%)	75	(45.2)	8	(36.4)	67	(46.5)	0.796	0.372^a^
Panic disorder, *n* (%)	3	(1.8)	1	(4.5)	2	(1.4)		0.349^c^
Separation anxiety disorder, *n* (%)	5	(3.0)	0	(0)	5	(3.5)		1.000^c^
Social phobia, *n* (%)	36	(21.7)	3	(13.6)	33	(22.9)		0.414^c^
Generalized anxiety disorder, *n* (%)	43	(25.9)	6	(27.3)	37	(25.7)	0.025	0.875^a^
Obsessive-compulsive disorder, *n* (%)	10	(6.0)	1	(4.5)	9	(6.3)		1.000^c^
Post-traumatic stress disorder, *n* (%)	3	(1.8)	0	(0)	3	(2.1)		1.000^c^
Specific phobia, *n* (%)	22	(13.3)	1	(4.5)	21	(14.6)		0.314^c^
Enuresis, *n* (%)	6	(3.6)	0	(0)	6	(4.2)		1.000^c^
ADHD, *n* (%)	44	(26.5)	7	(31.8)	37	(25.7)	0.367	0.544^a^
Disruptive behavior disorder, *n* (%)	42	(25.3)	10	(45.5)	32	(22.2)	5.450	**0.020^a^**
Oppositional defiant disorder, *n* (%)	39	(23.5)	10	(45.5)	29	(20.1)	6.805	**0.009^a^**
Conduct disorder, *n* (%)	12	(7.2)	3	(13.6)	9	(6.3)		0.200^c^
Tic disorders, *n* (%)	22	(13.3)	2	(9.1)	20	(13.9)		0.741^c^
Chronic motor or vocal tic disorder, *n* (%)	14	(8.4)	2	(9.1)	12	(8.3)		1.000^c^
Tourette’s disorder, *n* (%)	8	(4.8)	0	(0)	8	(5.6)		0.599^c^
Eating disorders, *n* (%)	12	(7.2)	0	(0)	12	(8.3)		0.370^c^

*Statistically significant differences (p < 0.05) are indicated in bold. ADHD, attention-deficit/hyperactivity disorder; IBC-R, Infant Behavior Checklist-Revised; K-SADS-PL, the Kiddie Schedule for Affective Disorder (Schizophrenia Present and Lifetime version); SD, standard deviation.*

*^a^Pearson’s χ^2^ test.*

*^b^Mann–Whitney U test.*

*^c^Fisher’s exact test.*

Table 2 shows the CBCL scores (total, internalizing, and externalizing scores as well as the scores for the eight subscales) in the chronic irritability and clinical control groups. The total and externalizing scores were significantly higher in the former than in the latter group (69.1 vs. 64.5, *p* = 0.046; 66.4 vs. 58.6, *p* = 0.001). Regarding the CBCL subscales, three of the eight subscale scores (attention problems, delinquent behavior, and aggressive behavior) were significantly higher in the chronic irritability group than in the clinical control group (66.6 vs. 61.4, *p* = 0.027; 63.4 vs. 58.5, *p* = 0.005; 66.0 vs. 59.2, *p* = 0.000).

**TABLE 2:** CBCL T scores for the chronic irritability and clinical control groups.

	Chronic irritability group		Clinical control group		t/U	*p*
	(*n* = 22)		(*n* = 144)			
**CBCL T scores, means (SD)**						
Problems (total)	69.1	(9.9)	64.5	(10.1)	2.008	**0.046^a^**
Internalizing	67.4	(9.6)	65.5	(11.1)	1,488.0	0.647^b^
Externalizing	66.4	(8.8)	58.6	(11.0)	856.5	**0.001^b^**
Withdrawal	66.5	(11.8)	64.6	(10.2)	1,455.0	0.537^b^
Somatic complaints	63.6	(8.5)	62.6	(10.8)	1,468.0	0.577^b^
Anxiety/depression	65.4	(12.3)	64.8	(11.3)	1,525.5	0.780^b^
Social problems	65.4	(10.8)	60.8	(9.1)	1,174.5	0.050^b^
Thought problems	66.6	(14.8)	60.5	(11.7)	1,213.5	0.067^b^
Attention problems	66.6	(11.1)	61.4	(8.7)	1,122.0	**0.027^b^**
Delinquent behavior	63.4	(8.1)	58.5	(8.6)	1,011.0	**0.005^b^**
Aggressive behavior	66.0	(9.1)	59.2	(9.2)	846.5	**0.000^b^**

*Statistically significant differences (p < 0.05) are indicated in bold.*

*CBCL, Child Behavior Checklist; SD, standard deviation.*

*^a^Student’s t-test.*

*^b^Mann–Whitney U test.*

Table 3 shows the comparative prevalence of SPD and the SSP scores (total scores as well as the sub-scores for the seven sections) in the chronic irritability and clinical control groups. The prevalence of SPD and the total SSP scores were significantly higher in the chronic irritability group than in the clinical control group (36.4 vs. 14.6%, *p* = 0.029; 76.5 vs. 59.7, *p* = 0.010). With regard to the SSP sections, five of the seven section scores (tactile sensitivity, movement sensitivity, underresponsive/seeks sensation, auditory filtering, and visual/auditory sensitivity) were significantly higher in the chronic irritability group than in the clinical control group (13.5 vs. 10.0, *p* = 0.004; 5.0 vs. 3.9, *p* = 0.015; 13.2 vs. 9.8, *p* = 0.002; 15.1 vs. 11.8, *p* = 0.016; 9.8 vs. 6.8, *p* = 0.001).

**TABLE 3:** Comparison of the prevalence of SPD and Short Sensory Profile scores between the chronic irritability and clinical control groups.

	Chronic irritability group		Clinical control group		χ^2^/U	*p*
	(n = 22)		(n = 144)			
Sensory processing difficulties, n (%)	8	(36.4)	21	(14.6)		**0.029^a^**
**SSP scores, mean (SD)**						
Total	76.5	(32.4)	59.7	(21.0)	1,045.5	**0.010^b^**
Tactile sensitivity	13.5	(6.6)	10.0	(4.1)	1,001.5	**0.004^b^**
Taste/smell sensitivity	7.7	(4.8)	6.1	(3.6)	1,206.0	0.053^b^
Movement sensitivity	5.0	(2.7)	3.9	(1.7)	1,142.0	**0.015^b^**
Underresponsive/seeks sensation	13.2	(5.6)	9.8	(4.7)	958.5.0	**0.002^b^**
Auditory filtering	15.1	(6.5)	11.8	(5.6)	1,081.0	**0.016^b^**
Low energy/weak	12.1	(6.2)	11.2	(6.3)	1,375.0	0.309^b^
Visual/auditory sensitivity	9.8	(5.5)	6.8	(3.2)	974.0	**0.001^b^**

*Statistically significant differences (p < 0.05) are indicated in bold. SD, standard deviation; SPD, sensory processing difficulties; SSP, Short Sensory Profile.*

*^a^Fisher’s exact test.*

*^b^Mann–Whitney U test.*

Table 4 shows the results of the forced entry multiple regression analysis. The total SSP scores were predicted by chronic irritability (β = 0.175; *p* = 0.007), age at consultation (β = -0.166; *p* = 0.012), low income (β = 0.202; *p* = 0.002), and internalizing CBCL scores (β = 0.400; *p* < 0.001).

**TABLE 4:** Results of multiple regression analysis showing predictors of SSP total scores in the chronic irritability group.

Variable	B	SE	β	*p*	95% CI (B)		VIF
Constant	1.070	12.822		0.934	–24.255	26.394	
Chronic irritability	12.044	4.419	0.175	**0.007**	3.316	20.772	1.095
Age (year)	–1.507	0.591	–0.166	**0.012**	–2.674	–0.339	1.120
Gender, male	1.117	3.080	0.024	0.717	–4.965	7.200	1.133
Low income	11.199	3.496	0.202	**0.002**	4.294	18.103	1.052
IBC-R score	0.514	0.384	0.087	0.182	–0.243	1.272	1.108
Internalizing T scores (CBCL)	0.857	0.153	0.400	**0.000**	0.556	1.159	1.345
Externalizing T scores (CBCL)	0.263	0.154	0.124	0.089	–0.041	0.568	1.393

*Statistically significant differences (p < 0.05) are indicated in bold. R^2^ = 0.374; Durbin–Watson score = 1.892.*

*β, standardized partial regression coefficient; B, unstandardized coefficient; CBCL, Child Behavior Checklist; CI, confidence interval; IBC-R, Infant Behavior Checklist-Revised; SE, standard error; SSP, Short Sensory Profile; VIF, variance inflation factor.*

## Discussion

To the best of our knowledge, our study is the first to demonstrate an association between chronic irritability and SPD in clinically referred children and adolescents. Our results suggest two points. First, approximately 13% of the enrolled children presented with chronic irritability. We note that children with chronic irritability often have ODD as well as externalizing and attention problems. Second, children with chronic irritability are more likely to have severe SPD.

As mentioned above, irritability is one of the most common reasons for children and adolescents to be referred for psychiatric evaluation and care (1). Prior studies have reported that the prevalence of irritability varies widely depending on the definition of irritability and the target population. For example, Toohey et al. reported an extremely high prevalence of irritability (99.3%). However, this value was obtained from asking adults in a community population if they had ever felt irritable during their lifetime (10). In contrast, Althoff et al. reported a very low prevalence of irritability (0.12%) when defining irritability based on a combination of frequency, severity, and period data in a community population of children aged 13–18 years (11, 20). This considerable variation in estimates might result from differences within the studies in terms of the frequency of outbursts, duration of irritability, and extent to which these outbursts and moods adhere to the DSM-5 criteria for dysfunction (excluding individuals with mania or hypomania) (50, 51).

To our knowledge, few studies have investigated the prevalence of irritability in clinical samples. The prevalence of six-month chronic irritability presenting with temper outbursts was 26–31% among the evaluated 6–12-year-old children who had been referred to a child psychiatric outpatient clinic (52, 53). This study defined chronic irritability as a severe form of irritability that persisted for a minimum of 1 year in at least two settings. Considering the duration (1 year of irritability) and the inclusion of adolescents with less severe irritability, our primary result (which was only slightly lower than that produced in the abovementioned report) is generally comparable to that reported in previous studies conducted in a clinical setting.

We showed that children with chronic irritability are more likely to have ODD and externalizing problems but are not more likely to have depression, anxiety disorders, or internalizing problems. With regard to DBD, ODD, and externalizing problems, our results are consistent with those of previous studies showing that irritability may be a dimension of ODD (4) and that childhood irritability significantly predicts DBD (54, 55). However, our results concerning internalizing problems, including depression and anxiety, are inconsistent with those of some prior studies conducted in adolescents that have shown associations between irritability and internalizing symptoms (5, 7). One of the potential reasons for this inconsistency is that our participants were younger than those enrolled in prior studies. For example, Copeland et al. have shown that irritability in childhood predicts depression in adulthood more strongly than the presence of a childhood depressive disorder does (56). Other studies have also reported that children with irritability are more likely to have problems with externalization, depression, and anxiety when evaluated longitudinally (21, 55, 57). Thus, our participants might have developed internalizing disorders over the course of several years. In addition, our study showed an association between chronic irritability and attention problems, consistent with the findings of previous research demonstrating that children and adolescents with chronic irritability have attentional impairments in the context of frustrative non-rewards (58).

To the best of our knowledge, this study is the first to show that children with chronic irritability are more likely to develop severe SPD. However, only a few studies have examined the association between chronic irritability and SPD. Benarous et al. (22) showed that children with DMDD, which presents with both chronic irritability and temper outbursts (as based on the diagnostic criteria), scored higher than typically developing children in standardized tests provided by the Sensory Profile manual, thus suggesting an association between chronic irritability and SPD. However, these studies reported no differences in Sensory Profile scores between children with and without DMDD in clinically referred children and adolescents. Therefore, the effect of chronic irritability on SPD remains unclear.

Contrastingly, the present study suggests that children with chronic irritability have more severe SPD in clinical settings. One potential reason for this discrepancy is the differences in participant characteristics and presentations between studies. For example, temper outbursts (one of the two core features of DMDD) may present rapidly increasing tantrums with episodic characteristics. This is in contrast to the tonic characteristics of chronic irritability. Cardinale (15) suggested that temper outbursts and chronic irritability might be distinct, although highly related, irritability dimensions. Second, the participants in the study by Benarous displayed suicidal behaviors, possibly making it difficult to obtain information on the effects of irritability from these subjects. Chronic irritability may have a more direct effect on SPD than temper outbursts. Further, this study demonstrated that chronic irritability is associated with more severe overall SPD, even after adjusting for IBC-R scores, internalizing and externalizing problems, age, sex, and low income. Some previous studies have found that SPD is more prevalent in children with internalizing (27) and externalizing problems (29) and in those of lower age, male sex, and lower socioeconomic status (27–29, 36, 59). Based on these studies, we conclude that the association between chronic irritability and SPD, which was detected in this study after adjusting for possible confounding factors, is reliable. Although the causal relationship between chronic irritability and SPD remains unknown, we conclude that SPD may facilitate emotional dysregulation, thus leading to the manifestation of chronic irritability in children (22). In this study, only the low energy/weak section showed no association with chronic irritability; this finding is consistent with the findings reported by Benarous (22). As the low energy/weak section is associated with poor muscle development and postural control (60), it is less strongly associated with irritability than the other sections. Irritability and SPD may share a common neurobiological basis. The pathophysiology of irritability comprises a background of aberrant reward processing, deficits of reward learning and prediction error, and cognitive control deficits (58, 61–64), indicating dysfunction in the prefrontal cortex, striatum, anterior cingulate, amygdala, and middle frontal gyrus (64–66). On the other hand, the integration of information from different sensory modalities is termed multisensory processing (67). Multisensory processing is associated with functions of the anterior cingulate, middle and inferior frontal gyri, and hippocampus (68, 69). Although no neurobiological studies in humans have examined the association between irritability and SPD, this association may be explained in future studies with reference to a common neurobiological basis as described above.

Our study has several limitations. First, we did not exclude participants with ASD, who often have sensory abnormalities (37). Moreover, we employed the K-SADS-PL DSM-IV-TR because the K-SADS-PL DSM-5 has yet to be translated to Japanese. ASD as a comorbidity may be a confounding factor that could influence the observed associations between chronic irritability and SPD. However, this association remained significant even after adjusting for IBC-R scores, which reflect the severity of ASD traits, thus reducing the possibility of confounding. We highly recommend that future studies use diagnostic tools for ASD, such as the Autism Diagnostic Interview (Revised). Second, this study included clinically referred children from a single university hospital, thereby warranting careful interpretation with regard to the generalization of our results to all children with chronic irritability. Third, the semi-structured interview for chronic irritability we developed was not completely validated. It was only assessed as a categorical variable and not as a continuous variable. Further, this study did not have a large sample of children with chronic irritability. However, we examined the inter-rater and test-retest reliability as well as the content validity by integrating information obtained from the children’s teachers. In fact, the current definitions of irritability and its related constructs have often been used interchangeably and non-rigorously in past studies (70–72). Unfortunately, there are no well-validated Japanese versions of continuous measures of irritability such as the Affective Reactivity Index. In the future, it is recommended that diagnostic tools for irritability such as the Affective Reactivity Index be used (3, 9). Fourth, our sample size is small, leading to the possibility of sampling bias and lack of statistical power. As noted above, this sample included children referred to the psychiatric outpatient clinic of the university hospital. Regarding the strength and robustness of our results, comorbidities were assessed in this study based on definite criteria using validated semi-structured interviews. Since we performed the Short Sensory Profile and assessed SPD as continuous variable, there were certain contributions to the final analysis.

In conclusion, this study provides evidence supporting our initial hypothesis that chronic irritability is associated with SPD in both children and adolescents. Since children with chronic irritability may often have SPD, it is necessary to assess SPD in order to administer appropriate interventions to children with chronic irritability. Additional longitudinal studies are needed to clarify the underlying mechanisms and to determine whether SPD develops into chronic irritability. We believe that our findings will guide future research directions and, if confirmed, ultimately inform clinical guidelines.

## Data Availability Statement

The original contributions presented in the study are included in the article/supplementary material, further inquiries can be directed to the corresponding author/s.

## Ethics Statement

The studies involving human participants were reviewed and approved by Ethical Committee of Osaka City University Graduate School of Medicine. Written informed consent to participate in this study was provided by the participants’ legal guardian/next of kin.

## Author Contributions

DM and YH were involved in the conception and design of the study and wrote the original draft. DM, YH, AG, KH, SS, HH, SK, and SN conducted data curation. DM, YH, AG, KH, SS, HH, SK, and KI were involved in the analysis, validation, or interpretation of data. All authors substantially revised the report for important intellectual content, improved the manuscript drafts, approved the final report, and accepted responsibility for all parts of this study to ensure that any questions regarding their accuracy or completeness are properly investigated and resolved.

## Conflict of Interest

The authors declare that the research was conducted in the absence of any commercial or financial relationships that could be construed as a potential conflict of interest.

## Publisher’s Note

All claims expressed in this article are solely those of the authors and do not necessarily represent those of their affiliated organizations, or those of the publisher, the editors and the reviewers. Any product that may be evaluated in this article, or claim that may be made by its manufacturer, is not guaranteed or endorsed by the publisher.
